# Exsection of the Entire Fibula for Fibro-Cartilaginous Degeneration of the Bone

**Published:** 1859-02

**Authors:** 


### ECLECTIC DEPARTMENT,

### AND SPIRIT OF THE MEDICAL PERIODICAL PRESS.

---

*Exsection of the Entire Fibula for Fibro-Cartilaginous Degeneration of the Bone.* By A. Reeves Jackson, M. D., of Stroudsburg, Pa.

It is believed that the following case is the only one on record, in which either of the bones of the leg has been *entirely* excised. It is remarkable, also, as exemplifying with regard to the lower extremity what has been shown by recent operations of Prof. J. M. Carnochan, published in previous numbers of this journal, to be true, respecting the bones of the forearm, viz: that in some instances the long bones may be entirely removed without serious loss of function of the limb and corresponding joints.

In the leg, it is commonly the tibia that forms the subject of such diseases as require excision; and as that bone is the one that enters into the formation of the knee and ankle joints, and upon whose broad, articulating surfaces the weight of the body is sustained, obviously, the entire excision of it could not be performed without leaving the patient a hopeless cripple, with a perfectly useless limb, and in a far worse condition than after amputation.

The present case, however, shows most conclusively that the fibula, which is comparatively an unimportant bone, may, when so seriously and extensively diseased as to demand it, be entirely removed, and the patient left with a useful member, whose functions are but slightly impaired.

*History.* Mrs. R., thirty-seven years of age, the wife of a farmer, and the mother of four children, noticed, some time during the month of May, 1849, a painless swelling of the outer side of the calf of the right leg, about four or five inches below the knee joint. The enlargement slowly increased, and extended itself upwards and downwards towards the knee and ankle joints. The right foot and lower part of the leg became œdematous, and she walked with a slight limp, which she attributed to weakness of the part. Her general health continued unimpaired. She attended to her ordinary household duties until the early part of September, when, in stepping down stairs, she felt "something give way," and fell to the bottom. She was now unable to walk, and for the first time applied for medical assistance.

Dr. G. A. Kaski, of Bartonsville, Pa., was sent for, who, after attendin the case a few days, and ascertaining its unusual character, requested my attendance in consultation.

Accordingly, we saw her together on the 19th September. On examination, we found the leg much swollen, painful on pressure, and the foot considerably everted. There seemed to be evident fracture of the fibula, although not the least crepitus could be produced. The leg was lightly dressed with splints and bandages, and cooling applications ordered to be applied.

I saw nothing more of the case for nearly three months, when I was again requested by Dr. Kaski to see her.

At this time, (Dec. 10th) the leg was much more enlarged, and the patient complained more of pain. The splints and bandages had been dispensed with for several weeks, owing to the irritation of the integuments which they produced. The eversion of the foot was still more marked than before. The general condition of the patient was, indeed, lamentable. She was greatly emaciated, although she did not present the appearances of a person suffering from malignant disease. Extensive ulcerations had occurred over the hips and sacrum, resulting from her long confinement to bed, and the discharges from which had seriously drained her system. There was much œdema of the parts about the ankle, foot, and on the fibular aspect of the leg. No fluctuation could be detected at any point.

Her condition was now such as to render it clear that something must be done for her relief, or that she would sink from exhaustion. Amputation of the limb had already been suggested to her, but to this measure the patient, as well as her friends, firmly objected. Excision of the affected bone was now proposed, and the nature of the operation having been explained to her, she consented to have it done. It was, accordingly, decided to excise the bone on the following Monday. In the mean time, she was put upon a course of tonic treatment, and the use of the most nourishing diet enjoined upon her.

*Operation.* The operation was performed December 22, 1849, in the presence, and with the assistance of Drs. Kaski and Foss, and Messrs. Kübler and Shick.

The patient having been put fully under the influence of chloroform, the leg was partly flexed, and a longitudinal incision made, commencing about a half inch above, and an inch in front of the head of the fibula, and extended downward to the external malleolus, dividing the skin and peroneii muscles. A second incision, starting from the same point as the first, was made transversely, and carried directly backwards about two inches. The flap thus formed was rapidly dissected off from the bone (or rather what was formerly bone) until the upper four inches were fully exposed. I now made an attempt to detach the head of the bone from its tibial articulation, but found it a very difficult proceeding. However, the substance representing the fibula, flexible and much thickened, was finally separated by passing a narrow-bladed bistoury between it and the tibia, the edges of the wound being held widely apart, at the same time, by an assistant with blunt hooks. Great care was necessary at this stage of the proceedings in order to avoid wounding the anterior tibial nerve, which was here seen passing down.

The upper portion, being now detached, was drawn outwards and made use of as a lever to aid in separating the remainder. Seizing it with the left hand for this purpose, ths fibres of the peroneus longus and the interosseous ligament were divided, the knife being kept close to the bone. In this manner, it was also separated from its connections with the soleus and the flexor longus pollicis pedis. Proceeding downwards, I found the attachments of the muscles and the interosseous ligament to the bone were so slight about its middle third, that they were readily separated by the handle of the knife. At one point there was scarcely any vestige of bone remaining.

The most difficult part of the operation consisted in removing the lower end of the bone from its attachments to the fasciculi of the external lateral ligiment of the ankle joint and the several short ligamentous attachments to the tiba. It was finally accomplished, however, by making a third incision, perpendicular to the first at its lower end, about an inch and a half long, and dissecting the flaps carefully back; then by pulling the diseased mass strongly outward, sufficient space was obtained for dividing the connection with a slender knife. Care was taken to avoid injuring the small slip of synovial membrane, which is here sent up, between the tibia and fibula, from the ankle joint.

The tibia and astragalus were both found to be unaffected by disease.

There was very little arterial hemorrhage throughout the operation. The peroneal artery, which was the only one of great size that was really in danger, was carefully avoided. Some of the anastomosing branches of the anterior and posterior peroneal arteries were cut, but torsion being applied to them, the bleeding soon ceased. The venous and capillary bleeding was troublesome for a time, but finally ceased, under the application of pressure, and the use of cold water.

*Dressing and Progress of Cure.* The edges of the wound were brought together, and retained by several points of interrupted suture and adhesive straps, the whole being covered lightly with a roller bandage. A well padded splint, four inches wide, and extending from a short distance above the knee joint to four or five inches below the foot, was placed along the tibial aspect of the limb, and confined by a few turns of the roller above the knee and to the foot, in such a manner as to draw the sole of the foot more nearly to its natural position. Cold water dressing was then ordered, to be kept constantly applied to the wound. A full dose of morph. sulph. was given at bedtime.

The sutures were removed on the third day, at which time union was found to have occurred throughout the greater part of the wound. The patient was kept upon the use of tonics, and a full diet allowed. The subsequent progress of the case was entirely satisfactory; and at the end of three weeks the patient was able to sit upon a chair, with the foot and leg resting upon an elevated cushion. In two and a half months, she was able to walk with a cane, by the aid of a stout gaiter-shoe, to the sole of which was attached a steel plate, three-fourths of an inch wide, and one-eighth of an inch thick, which, being applied to the outer side of the limb, extended upwards, to a point opposite the tibio-fibular articulation, and the upper end, being well padded, was secured by a broad strip of ferreting, passed around the leg, below the knee. This apparatus she used for about two years, when she at length threw it aside, and ever since has merely used a cane.

*Functions of the Leg and Foot.* She walks with a slight limp, bearing the weight of the body upon the inner side of the foot, the sole of which is considerably everted. Owing to the detachment of the biceps muscle, she has no power to evert the leg, when in a semi-fixed position. The soleus, however, continues to act from its tibial attachment. There is a tendency in the foot to be partially flexed from the detachment of the peroneus longus and peroneus brevis, those muscles being extensors of the foot, and antagonistic to the tibialis anticus and peroneus tertius, which flex the foot. The motion at the ankle joint is somewhat impaired, owing, perhaps, to the long continued eversion of the foot, and the consequent side pressure on the joint.

*Appearance and Pathological Condition of the Bone.* It is thickened throughout its entire extent, but most so about the middle and upper extremity. At the largest point it measures five and three-quarter inches in circumference. It is of a yellowish-white color, in consistence rather softer than cartilage, and thickly studded with osseous spiculæ, imbedded in a dense, elastic, fibrous tissue. Its external surface is rough and irregular, and presents numerous small cavities of varying size. The periosteum is entirely removed from the posterior surface of the upper half of the bone, and although still present on some parts of the anterior surface, it is much thickened and softened. At one point there is no bone whatever, for a distance of an inch and a half, the upper and lower extremities being held together merely by a few shreds of periosteum. The lower part of the bone is tolerably firm, enlarged to about twice its normal size, and the periosteal investment unaltered. The interior of the bone, however, is softened, and degeneration of the tissue considerably advanced.—*American Journal Med. Sciences.*

---

Amongst the printed communications, forwarded to Academy, Mr. Flourens noticed a pamphet by Mr. Martini, on the Effects of Santonine upon Vision.

Santonine (a crystalized and bitter substance, obtained from various kinds of Artemisias,) is possessed of this singular property, that a few minutes after being taken, it causes all objects to appear green. This color, according to Mr. Martini, is not the same for all persons, and varies, according to the doses of santonine. A person to whom this substance had been given as an anthelmintic, stated, twenty minutes after taking the dose, that surrounding objects appeared deep green, whereas to one of the Professor's pupils they seemed of a blue color. A young man, upon whom five grains of santonine produced the effect of spreading a yellow color over surrounding objects, thirty-six minutes after taking ten grains, saw them red, half an hour later, orange color, and again yellow. In no instance did this singular phenomenon last more than twenty-four hours.

Mr. Martini's experiments led to two other communications on the same subject by Messrs. Leroy d'Etiolles and Mialhe. Mr. Leroy stated that two children, to whom he had exhibited santonine, for worms, excreted green colored urine. Mr. Mialhe brought forward cases confirming Mr. Leroy's remark, and attributed the phenomenon to the oxidation of the santonine in the circulation.—*Jour. Pract. Med. and Surg.*

